# COVID-19 impact on health service- and TB-related practices among private providers in Indonesia

**DOI:** 10.5588/pha.23.0056

**Published:** 2023-06-21

**Authors:** B. W. Lestari, A. Alifia, F. N. Soekotjo, Hariyah, A. F. Sumantri, I. D. Kulsum, B. Alisjahbana

**Affiliations:** 1 Department of Public Health, Faculty of Medicine, Universitas Padjadjaran, Bandung, Indonesia; 2 Tuberculosis Working Group, Faculty of Medicine, Universitas Padjadjaran, Bandung, Indonesia; 3 Indonesian Medical Association – Bandung Chapter, Bandung, Indonesia; 4 TB Supervisor, Provincial Health Office of West Java, Bandung, Indonesia; 5 Department of Internal Medicine, Faculty of Medicine, Universitas Islam Bandung, Bandung, Indonesia; 6 Department of Internal Medicine, Dr Hasan Sadikin General Hospital, Bandung, Indonesia; 7 Indonesian Professional Organisation Coalition for Tuberculosis – Bandung Chapter, Bandung, Indonesia

**Keywords:** health service delivery, tuberculosis, private healthcare providers

## Abstract

**SETTING:**

The COVID-19 pandemic has caused disruptions to healthcare services worldwide, including in private healthcare facilities (HCFs), where TB patients mostly initiate their care-seeking journey.

**OBJECTIVE:**

To identify adjustments to TB-related practices made by HCFs during the pandemic.

**DESIGN:**

We identified, contacted and invited private HCFs across West Java, Indonesia, to fill an online questionnaire. The questionnaire explored participants’ sociodemographic characteristics, adaptations and TB management practices implemented in their facilities during the pandemic. Data were analysed using descriptive statistics.

**RESULTS:**

Of the 240 HCFs surveyed, 40.0% shortened their operational hours and 21.3% have ever closed their practices during the pandemic; 217 (90.4%) made adjustments to keep delivering services, 77.9% by requiring the use of personal protective equipment (PPE); 137 (57.1%) observed fewer patient visits; 140 (58.3%) used telemedicine, a few of which (7.9%) ever handled TB patients on that platform. Respectively 89.5%, 87.5% and 73.3% of HCFs referred patients for chest radiography, smear microscopy and Xpert testing. Only a median of 1 (IQR 1–3) TB patient per month was diagnosed by the HCFs.

**CONCLUSION:**

Two major adaptations rolled out during COVID-19 were the use of telemedicine and PPE. Optimisation of the diagnostic referral system to increase TB case detection in private HCFs is warranted.

## Abstract (fr)

**CADRE:**

La pandémie de COVID-19 a perturbé les services de santé dans le monde entier, y compris dans les établissements de santé (HCF) privés, où les patients atteints de TB entament généralement leur parcours de recherche de soins.

**OBJECTIF:**

Identifier les ajustements des pratiques liées à la TB réalisés par les HCF pendant la pandémie.

**CONCEPTION:**

Nous avons identifié, contacté et invité des HCF privés à travers Java occidental, en Indonésie, à remplir un questionnaire en ligne. Le questionnaire a exploré les caractéristiques sociodémographiques des participants, les adaptations et les pratiques de gestion de la TB mises en place dans leurs établissements pendant la pandémie. Les données ont été analysées à l’aide de statistiques descriptives.

**RÉSULTATS:**

Sur les 240 HCF interrogés, 40,0% ont réduit leurs heures d’ouverture et 21,3% ont fermé leurs pratiques pendant la pandémie ; 217 (90,4%) ont apporté des ajustements pour continuer à fournir des services, 77,9% en exigeant l’utilisation d’équipements de protection individuelle (PPE) ; 137 (57,1%) ont observé moins de visites de patients ; 140 (58,3%) ont utilisé la télémédecine, dont seulement quelques-uns (7,9%) ont traité des patients atteints de TB sur cette plateforme. Respectivement, 89,5%, 87,5% et 73,3% des HCFs ont référé les patients pour une radiographie pulmonaire, une microscopie des crachats et un test Xpert. Seul un nombre médian de 1 (intervalle interquartile 1–3) patient atteint de TB par mois a été diagnostiqué par les HCF.

**CONCLUSION:**

Deux adaptations majeures ont été déployées pendant la COVID-19 : l’utilisation de la télémédecine et des PPE. Une optimisation du système de référence diagnostique pour augmenter la détection des cas de TB dans les HCFs privés est justifiée.

Healthcare services worldwide, including those addressing TB, have been extensively disrupted by the emergence of the COVID-19 pandemic.^1,2^ In 2020, the WHO predicted that interruptions to TB service delivery might be as high as 25–50%, resulting in an extra 63 million cases of TB and 14 million TB-related deaths between 2020 and 2025.^3,4^ This was followed by a decrease in TB notification rates and an increase in TB deaths, resulting in a 5-year delay in TB mortality and a 9-year delay in TB detection.^5^ The current situation is alarming as TB had already surpassed all other infectious agents to become the number one cause of death globally, even before the onset of the COVID-19 pandemic.^4^ According to the WHO, an estimated 14 million fewer people (21% fewer) got TB care in 2020 compared to 2019, and Indonesia, the second highest TB burden country, had the second largest shortfall in TB notifications worldwide in 2020 compared to 2019.^1,4^ TB referrals for people with suspected TB also declined by 59%. These indicate that most of the progress gained in detecting TB patients during the last decade was reversed in 2020.^6,7^

Health services have been adversely affected by the continuation of COVID-19 mitigation measures and the redirection of resources in response to the pandemic, resulting in a reduction in their capacity to deliver preventive and treatment services for various illnesses, including TB.^2,6^ These interruptions not only affected public health facilities but also the private sector, which is usually the initial point of care for patients.^8^ A patient pathway study showed that 76.8% of patients sought their first care in private healthcare facilities (HCFs).^9^ Nevertheless, the monitoring and control of private HCFs frequently fall beyond the scope of national TB programmes.^8^

Even before the pandemic surfaced, the integration between the public and private sectors in TB care programmes had been a struggle, particularly in low-resource, high-burden TB settings.^8^ A patient pathway analysis in 2017 in Indonesia found that while private providers contributed 42% to TB management, they only contributed 9% to case notifications.^10^ Hence, although TB diagnostic and treatment services are mostly offered through the public health system, the private sector is a crucial entry point for people seeking healthcare.^9^ To expedite case identification and treatment success and address the problem of missing cases,^11,12^ the WHO has introduced a global policy for TB control that adopts a public-private mix (PPM) approach involving all service providers. Furthermore, engaging private providers (PPs) is crucial in mitigating unnecessary deaths and suffering arising from incorrect diagnosis and treatment, reducing transmission by minimising treatment delays, and accelerating the adoption of new tools.^13,14^

Understanding the significance of COVID-19 and how it has influenced TB in the private sector is crucial for the scale-up of the TB management system in future. Unfortunately, only a handful of studies from high TB burden settings have focused on the relevant themes. The purpose of this study is to determine the effects of COVID-19 on private HCFs, with an emphasis on their TB-related practices, in West Java - the Indonesian province with the highest TB burden.^15^

## METHODS

### Study design and setting

This cross-sectional study was conducted in West Java Province, Indonesia, from March to September 2022. The province, which supports 49.9 million inhabitants and comprises of 18 regencies and nine cities, is classified into six regions: Bodebek, Baraya, Purwasuka, Priangan Timur, Priangan Barat and Ciayu Majakuning. Healthcare services are provided by a range of public and private facilities; as of 2020, there were 1,093 community health centres (CHCs), 377 public and private hospitals, 3,770 private clinics and 11,988 private specialists and general practitioners across the province.^16^

### Study population

Health practitioners who work at private HCFs that provide healthcare services in Bodebek and Baraya regions were eligible for the study. These two regions were selected in consultation with the Provincial Health Office due to their high population density and large number of private healthcare providers.

We contacted private HCFs in the area and invited their healthcare workers (HCWs) to fill in an online questionnaire. Participating HCWs could be physicians, nurses or pharmacists. Participants who were employed by National Tuberculosis Programme (NTP) healthcare facilities were excluded from the data pool. Entries from participants whose facilities operated for less than 3 months at the time of data collection were also excluded. A single HCW was chosen to represent each HCF, with the selection based on their level of responsibility for TB patients and following this order of priority: 1) physicians, 2) nurses, 3) pharmacists.

The questionnaire was piloted to a small number of participants to ensure that the questions were understandable and contextually correct. The questionnaire gathered information on 1) demographic and professional background of the participants, 2) characteristics of their HCFs, and 3) disruptions experienced and adjustments made at their HCFs, including in TB-related practices.

### Analysis

Before data analysis, data cleaning such as validating source of information, checking for completeness, uniforming data format (e.g., months into years) and removing duplicates was also done by the study team. We used a descriptive statistical analysis for this study. Numeric variables are presented in mean and standard deviation (SD) if they are normally distributed, or in median and interquartile range (IQR) if they are not. Statistical analysis was done using SPSS v25 (IBM Corp, Armonk, NY, USA).

Ethical approval was given by Universitas Padjadjaran’s Ethical Committee; Bandung, Indonesia (Reg. No. 2207070810).

## RESULTS

### Demographic and professional backgrounds of study participants

A total of 361 questionnaire responses were collected, and 261 responses from 240 HCFs were included in the analysis; the majority of the participants were physicians (59.4%); of whom 81.6% worked in private clinics, 15.3% had solo practices, while respectively 2.3% and 0.8% worked in private hospitals and public non-NTP healthcare facilities; 75.5% of participants were female, and the median age of all participants was 37 years (IQR 29–45); participating physicians were older (median age: 42 years, IQR 32–50) and had accumulated more clinical experience (median: 14.3 years, IQR 5.5–21.5) than their counterparts. Many of the participants were untrained in the subject of TB management (60.2%) and public–private partnership (90.4%) (Table 1).

**TABLE 1:** Demographic and professional characteristics of study participants (*N* = 261)

Category	Physicians (*n* = 155, 59.4%) *n* (%)	Nurses (*n* = 83, 31.8%) *n* (%)	Pharmacists (*n* = 23, 8.8%) *n* (%)
Age, years, median [IQR]	42 [32–50]	32 [26–39]	32 [26–38]
Female	107 (69.0)	67 (80.7)	23 (100.0)
Duration of clinical experience, years, median [IQR]	14.3 [5.5–21.5]	9.0 [3.0–15.0]	6.0 [3.0–10.0]
Last education			
General practitioner	152 (98.1)	N/A	N/A
Specialist	3 (1.9)	N/A	N/A
Work station			
Private clinic	113 (72.9)	77 (92.8)	23 (100.0)
Solo practice	39 (25.2)	1 (1.2)	0 (0.0)
Private hospital	2 (1.3)	4 (4.8)	0 (0.0)
Public non-NTP healthcare facility	1 (0.6)	1 (1.2)	0 (0.0)
Member of professional organisation	152 (98.1)	77 (92.8)	23 (100.0)
TB training experience			
Trained within the past 5 years	41 (26.5)	26 (31.3)	1 (4.3)
Trained over the past 5 years	31 (20.0)	4 (4.8)	1 (4.3)
Untrained	83 (53.5)	53 (63.9)	21 (91.3)
Public-private mix training experience			
Trained within the past 5 years	14 (9.0)	9 (10.8)	0 (0.0)
Trained over the past 5 years	2 (1.3)	0 (0.0)	0 (0.0)
Untrained	139 (89.7)	74 (89.2)	23 (100.0)

IQR = interquartile range; N/A = not available; NTP = National TB Programme.

### Adjustments in practices during COVID-19 pandemic

Overall, 57.5% of the HCFs experienced fewer patient visits, while 52.1% underwent a shortage of medical supplies; 70.8% of the HCFs required use of personal protective equipment (PPE) to keep operating their services as regulated by the government; 67.1% of HCFs also responded to the pandemic by offering COVID-19 testing services, mainly using rapid antigen tests.

Only 21.3% of HCFs ever closed their practices during the pandemic, mostly due to lack of available staff. Around a third of all HCFs had to discontinue some services in their practice, mainly face-to-face consultation. Additionally, half of the HCFs provided telemedicine services to connect with patients during the pandemic (Table 2).

**TABLE 2:** HCF characteristics and its practices during COVID-19 pandemic (*N* = 240)

Category	*n* (%)
Characteristics of HCFs	
Location*	
Bodebek	130 (54.2)
Baraya	110 (45.8)
BPJS (NHI) collaboration	
Yes	168 (70.0)
No	72 (30.0)
Adjustments made during COVID-19 pandemic: closed practices (*n* = 51)	
Reasons of closing	
Lack of available staff^†^	29 (56.9)
Regulated safety protocol^‡^	14 (27.5)
Lack of PPE	7 (13.7)
Small number of patients	4 (7.8)
Changes in operational hours	
Shorter operational hours	96 (40.0)
Longer operational hours	7 (2.9)
No change in operational hours	137 (57.1)
Changes in patient visits	
Fewer visits	138 (57.5)
More visits	59 (24.6)
No change	34 (14.2)
Not answered	9 (3.8)
Discontinued services (*n* = 74)	
Face-to-face consultation^§^	25 (33.8)
Diagnostic services^¶^	24 (32.4)
Hospitalisation	10 (13.5
Special services^#^	4 (5.4)
Treatment services**	12 (16.2)
Implemented essential measures (*n* = 217)	
Require use of PPE^††^	170 (78.3)
Limit number of people in closed spaces^‡‡^	33 (15.2)
Limit daily number of patients	19 (8.7)
Raise service fees	3 (1.4)
Experienced disturbances (*n* = 174)	
Shortage in medical supplies	125 (71.8)
Shortage in human resources	34 (19.5)
Facility renovation for infection control	17 (9.7)
Raise of prices of medical supplies	1 (0.6)
Enforced government-mandated regulations (*n* = 110)	
Use of PPE	102 (92.7)
Reduce building capacity for patients and staffs	9 (8.2)
Pricing limits	6 (5.5)
Free COVID-19 services	5 (4.5)
Mandatory reporting of COVID-19 tests and cases	1 (0.9)
COVID-19-related practices	
COVID-19 services available	
Testing services only (rapid antigen)	114 (47.5)
Treatment services only	17 (7.1)
Both testing and treatment services	47 (19.6)
No COVID-19 related services	62 (25.8)
Offer COVID-19 tests for patients with respiratory symptoms (*n* = 230)	
Yes	143 (62.2)
No	87 (37.8)
Availability of telemedicine	
Yes	140 (58.3)
No	94 (39.2)
Not answered	6 (2.5)

*Classifications of the site locations: Bodebek = Bogor City, Depok City, Bekasi City, Bogor Regency, Bekasi Regency; Baraya = Bandung City, Cimahi City, Bandung Regency, West Bandung Regency, Sumedang Regency.

†General lack of staff, staff contracted COVID-19 and staff at high risk of contracting COVID-19.

^‡^Government-mandated large-scale social distancing and independently regulated clinic sterilisation procedure.

^§^General face-to-face consultation, teeth polyclinic.

^¶^Radiology services, physical examination, skin examination, blood/urine lab services, tuberculosis screening, tuberculosis testing, home visit.

^#^Maternal and child care services.

^**^Nebulisation, surgery.

^††^Mandatory personal protective equipment use for healthcare workers and/or patients.

^‡‡^Limitations on number of healthcare workers and/or patients in closed spaces in specified timeframe.

HCF = healthcare facility; BPJS = Badan Penyelenggara Jaminan Sosial. NHI = ­National Health Insurance; PPE = personal protective equipment.

### Management of tuberculosis-related practices in healthcare facilities across West Java

#### Healthcare facility-level analysis

Of all HCFs, respectively 95.0%, 90.0% and 73.3% chose chest X-ray (CXR), smear microscopy and Xpert testing to diagnose TB. Overall, 90.8% chose the combination of CXR and either smear microscopy or Xpert to obtain TB diagnosis.

Of those who opted for CXR, 92.1% referred their patients to other HCFs for the test, with 48.6% directing them to private hospitals or laboratories. A similar trend was observed among the 216 facilities that chose smear microscopy, with 99.1% of them opting to refer patients to other facilities for the test. Of these, 82.7% referred patients to CHCs, public laboratories or public hospitals for testing. Similarly, 100.0% of 176 facilities who chose Xpert also referred patients for the test, mainly to public HCFs (89.8%), such as CHCs, public laboratories and public hospitals (Figure).

**FIGURE:**
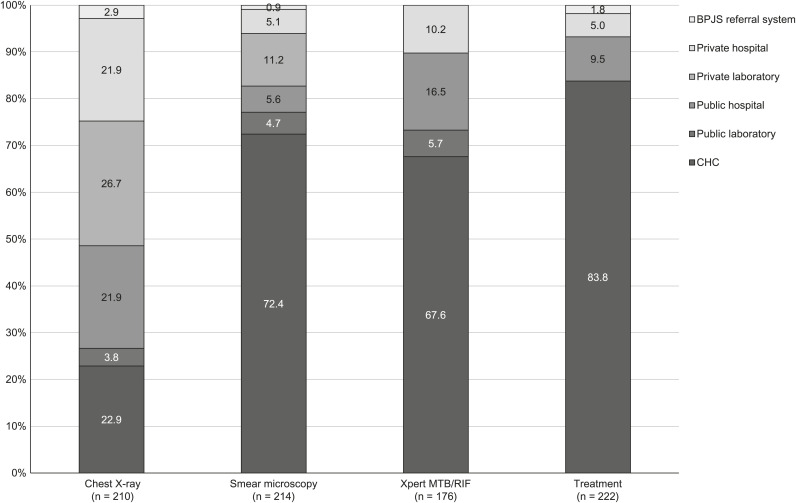
Number of TB referrals in all private healthcare facilities (total = 240; chest X-ray = 210; smear microscopy = 214; Xpert MTB/Rif = 176, those treated = 223). BPJS = *Badan Penyelenggara Jaminan Sosial*; CHC = community health centre.

Only 7.5% HCFs treated TB patients at their own HCFs, while 92.5% referred their patients to other HCFs, mostly to CHCs and public hospitals (93.2%) (Figure). The median number of monthly diagnosed and treated TB cases at each HCF was respectively 1 (IQR 1–3) and 1 (IQR 0–4). Only 56.7% of all HCFs were contacted by CHCs for TB case reporting.

#### Practitioner-level analysis

Of 261 HCWs, most of them were aware that it is mandatory to report TB cases to the government. However, almost a quarter of HCWs still felt uncomfortable complying with this guidance, mainly because of the complicated TB forms. Around half of all participants mentioned messaging applications as one of their preferred methods for TB case reporting. Only a few HCWs ever handled TB cases in telemedicine platform (Table 3).

**TABLE 3:** Management of TB-related practices in HCF across West Java

Category	*n* (%)
HCF-level analysis (*n* = 240)	
Admits patients with respiratory symptoms	
Yes	237 (98.8)
No	3 (1.3)
Number of handled TB cases at HCF	
Diagnosed TB cases/month, *n*, median [IQR]	1 [1–3]
Treated TB cases/month, *n*, median [IQR]	1 [0–4]
Contacted by CHC for TB case reporting	
Yes	136 (56.7)
No	104 (43.3)
Provider-level analysis (*n* = 261)	
Knowledge of mandatory TB cases reporting to government	
Yes	251 (96.2)
No	10 (3.8)
Feeling of comfort in reporting TB cases to government	
Yes	197 (75.5)
No	64 (24.5)
Reasons of discomfort (*n* = 64)	
Complicated forms	28 (43.8)
Do not have enough time	21 (32.8)
Often forgot, not routinely recorded	14 (21.9)
Need to keep patient data confidential	12 (18.9)
Other*	7 (10.9)
Preferred method of TB case reporting	
Messaging application	143 (54.8)
Written form	78 (29.9)
Smartphone application	70 (26.8)
Email/website	57 (21.8)
Direct reporting to CHC	5 (1.9)
Experience in handling TB cases using telemedicine (*n* = 247)	
Yes	11 (4.5)
Cases handled via telemedicine (*n* = 11)	
Screening of TB suspects, *n*, mean ± SD	1.2 ± 0.9
Consultation of newly diagnosed TB patients, *n*, median [IQR]	1.0 [0–1]
Treatment monitoring of TB patients, *n*, median [IQR]	1.0 [1–4]
TB diagnostic referral from telemedicine (*n* = 11)	
Refer to other HCF	7 (63.6)
Sputum delivery services	2 (18.2)
Home visit	1 (9.1)
Patients left without being tested	1 (9.1)
No	236 (95.5)
Confidence level in differentiating COVID-19 and TB	
Very confident	40 (15.3)
Confident	170 (65.1)
Somewhat confident	35 (13.4)
Not confident	7 (2.7)
Not answered	9 (3.4)

*Includes healthcare workers were not well-received by CHC, unknowing of the ­reporting procedure, and feel no use of reporting TB cases to the government.

HCF = healthcare facilities; IQR = interquartile range; CHC = community health centre.

## DISCUSSION

This study identifies health systems disruptions and adjustments made to private healthcare practices in West Java during the COVID-19 pandemic. Some private HCFs had to discontinue in-person consultations, and a significant portion of the HCFs experienced fewer patient visits. Regarding TB diagnosis, several HCFs referred their patients to other private and public HCFs for the necessary tests. Very few HCFs treated their TB patients independently. It was observed that not all HCFs were contacted by CHCs for TB reporting, and some HCWs were discouraged from reporting due to the complexity of the reporting forms. These challenges faced by HCFs impeded the identification and notification of TB cases during the pandemic.

In addition to the discontinuation of face-to-face consultations and fewer patient visits, we observed that there were many HCFs who started to deploy telemedicine during the pandemic. This is in line with other studies from the United States and India that reported increases in telemedicine use in healthcare providers triggered by the pandemic, especially in its early phase.^17,18^ However, although telemedicine offers many benefits for TB management, very few of our participants ever utilised telemedicine for TB cases.

A significant proportion of HCFs were found to lack TB diagnostic tests and instead opted to refer their patients to external service providers. Almost half of the HCFs referred their patients to other private HCFs for CXR examination, while for smear microscopy and Xpert tests, they primarily directed their patients to public HCFs. It was concerning to note that around a quarter of the participants did not include Xpert testing as a part of their TB diagnostic tools, despite it being a highly specific and sensitive TB test recommended by the Indonesian Ministry of Health in both the national TB guideline and the Minister’s Decree,^19,20^ and a total of 79 GeneXpert machines are readily available in various public facilities in Bodebek and Baraya regions.^21^ Due to the limited number of trained participants in both TB and public-private partnership subjects, it remains unclear whether the observed trend is a result of unfamiliarity with TB guidelines or a lack of comprehension of the Xpert referral scheme.

Most HCFs do not treat their TB patients by themselves and refer their patients to CHCs and public hospitals, although many of them have collaborated with Badan Penyelenggara Jaminan Sosial (BPJS), which should be beneficial in providing treatment. A study found that private practitioners were often reluctant to treat their patients, despite collaboration with BPJS,^12^ due to several reasons, including the possibility of increased transmission risk at their workplace, high drug cost, treatment complexity, human resource limitations and the feeling of being excluded from national TB efforts.^12^ While BPJS has been designed to address concerns regarding drug costs, there have been reports indicating that its full potential is not being utilised in private practices. One possible contributing factor to the limited participation of private healthcare facilities in the BPJS system could be the payment procedures established by BPJS. These procedures may inadvertently create disincentives for private facilities to fully engage with the system, ultimately hindering rather than facilitating progress towards the goal of affordable healthcare for all.^22^

Underreporting is also a persisting issue in TB case notification.^23^ Some of our participants feel uncomfortable in reporting TB cases, complicated forms being the main barrier reported. To note, a study conducted in India revealed that while some HCWs feel comfortable reporting TB cases, many of them fail to report it to the government.^24^ The reasons for this were cited as a lack of time and concerns regarding patient confidentiality, which were similar to the reasons reported by participants, who expressed discomfort in reporting such cases. Most of our participants preferred messaging applications such as SMS and WhatsApp group chat as a way to report TB cases; this is in line with findings from other studies.^18,25^ Additionally, around half of our participants also mentioned that in the last 6 months, they were not visited by CHCs officers for TB case reporting.

To the best of our knowledge, this is the first attempt to gather a substantial number of private practitioners across West Java, in order to document their experiences with the COVID-19 pandemic and its effects on general and TB-related practices. This study has several limitations that should be noted. First, relied on the recollections of practitioners regarding their experiences with the pandemic and how it impacted their services over the last two years. The changes observed were primarily categorized as subjective measures, such as shorter hours and an increase in patient visits. To assist participants in recollecting and organizing their experiences, the study presented various categorisation options. Second, as we only included participants from Bodebek and Baraya areas, the results might not be representative of other parts of the province. Third, it is possible that our study may have been affected by selection bias during the recruitment process, as practitioners with a higher level of awareness and knowledge about COVID-19 and TB may have been more inclined to participate.

From this study, several recommendations may be considered. First, the current wide use of telemedicine for COVID-19 can be utilised for TB care as well. TB screening methods for COVID-19 patients, including those using telemedicine, can be applied to increase TB detection.^2,26^ Second, the referral systems for TB diagnosis and treatment between public and private healthcare sectors require strengthening. A stronger effort is needed to ensure private practitioners to have a better understanding of these systems to increase patients’ access to TB diagnostics and treatment. Establishing a strong specimen referral system from private providers to central laboratories can help streamline pathways to care.^9^ To ensure optimal implementation, the government should also pay attention to patients’ understanding of acceptable specimen criteria and HCW skills in safely packaging specimens to avoid unusable or damaged specimens.^27,28^ Third, simpler TB reporting tools should be researched to increase case notifications from private providers. Furthermore, CHCs officers should monitor TB case recording and reporting from private providers regularly.^25,29^

## CONCLUSION

During the COVID-19 pandemic, a significant number of private HCFs began incorporating telemedicine and implementing the use of PPE in their operations. These changes have been observed among the majority of private HCFs that participated in this study.
